# Heavy-metal associated breast cancer and colorectal cancer hot spots and their demographic and socioeconomic characteristics

**DOI:** 10.1007/s10552-024-01894-0

**Published:** 2024-06-25

**Authors:** Madeline M. Tomlinson, Felicia Pugh, Alexandra N. Nail, Johnnie D. Newton, Karen Udoh, Stephie Abraham, Sandy Kavalukas, Brian Guinn, Rulla M. Tamimi, Francine Laden, Hari S. Iyer, J. Christopher States, Matthew Ruther, C. Tyler Ellis, Natalie C. DuPré

**Affiliations:** 1 Department of Epidemiology and Population Health, School of Public Health and Information Sciences, University of Louisville, 485 E Gray St, Louisville, KY 40202 USA; 2 Louisville Metro Department of Public Health and Wellness, Center for Health Equity, Louisville, KY USA; 3 Department of Pharmacology and Toxicology, School of Medicine, University of Louisville, Louisville, KY USA; 4 Department of Surgery, School of Medicine, University of Louisville, Louisville, KY USA; 5 Department of Population Health Sciences, Weill Cornell Medical, New York, NY USA; 6 Channing Division of Network Medicine, Department of Medicine, Brigham and Women’s Hospital and Harvard Medical School, Boston, MA USA; 7 Department of Epidemiology and Department of Environmental Health, Harvard TH Chan School of Public Health, Boston, MA USA; 8 Section of Cancer Epidemiology and Health Outcomes, Rutgers Cancer Institute of New Jersey, New Brunswick, NJ USA; 9 Center for Integrative Environmental Health Sciences, University of Louisville, Louisville, KY USA; 10 Department of Urban and Public Affairs, College of Arts and Sciences, University of Louisville, Louisville, KY USA

**Keywords:** Breast cancer, Colorectal cancer, Arsenic, Cadmium, Metals, Cancer registry

## Abstract

**Purpose:**

Cancer registries offer an avenue to identify cancer clusters across large populations and efficiently examine potential environmental harms affecting cancer. The role of known metal carcinogens (i.e., cadmium, arsenic, nickel, chromium(VI)) in breast and colorectal carcinogenesis is largely unknown. Historically marginalized communities are disproportionately exposed to metals, which could explain cancer disparities. We examined area-based metal exposures and odds of residing in breast and colorectal cancer hotspots utilizing state tumor registry data and described the characteristics of those living in heavy metal-associated cancer hotspots.

**Methods:**

Breast and colorectal cancer hotspots were mapped across Kentucky, and area-based ambient metal exposure to cadmium, arsenic, nickel, and chromium(VI) were extracted from the 2014 National Air Toxics Assessment for Kentucky census tracts. Among colorectal cancer (*n* = 56,598) and female breast cancer (*n* = 77,637) diagnoses in Kentucky, we used logistic regression models to estimate Odds Ratios (ORs) and 95% Confidence Intervals to examine the association between ambient metal concentrations and odds of residing in cancer hotspots, independent of individual-level and neighborhood risk factors.

**Results:**

Higher ambient metal exposures were associated with higher odds of residing in breast and colorectal cancer hotspots. Populations in breast and colorectal cancer hotspots were disproportionately Black and had markers of lower socioeconomic status. Furthermore, adjusting for age, race, tobacco and neighborhood factors did not significantly change cancer hotspot ORs for ambient metal exposures analyzed.

**Conclusion:**

Ambient metal exposures contribute to higher cancer rates in certain geographic areas that are largely composed of marginalized populations. Individual-level assessments of metal exposures and cancer disparities are needed.

**Supplementary Information:**

The online version contains supplementary material available at 10.1007/s10552-024-01894-0.

## Introduction

In the United States (US), breast cancer is the most common cancer diagnosed in women and colorectal cancer is the third most commonly diagnosed cancer, excluding non-melanoma skin cancer [1]. There is substantial variability in breast cancer and colorectal cancer across the US as well as cancer disparities across racial and ethnic groups, with highest rates of mortality from these cancers in Black populations [1–3]. Despite some of these regional differences being attributed to variation in known lifestyle risk factors [4, 5], epidemiologic studies suggest that environmental factors play a role in the development of breast cancer [6–10] and colorectal cancer [11–17]. Heavy metals and metalloids are significant contributors to toxic environmental pollutants. Heavy metals are naturally occurring elements in the earth’s crust, though human exposure happens most often from anthropogenic sources such as combustion, industrial processing, waste incineration, and waste sites [18–24]. Historically marginalized populations and those living in highly racially segregated neighborhoods were more likely to reside in areas with higher heavy metal exposure [25–31]. Environmental injustices may contribute to breast and colorectal cancer disparities.

Cadmium, arsenic, nickel, and hexavalent chromium are Group 1 human lung, skin, and/or bladder carcinogens [32]. The epidemiologic literature for these exposures in relation to breast or colorectal cancer risk was sparse at the time of the IARC panel review [23, 33]. These non-essential heavy metals drive biological mechanisms relevant to tumorigenesis, such as inducing reactive oxygen species, directly damaging DNA, inhibiting DNA double-strand break repairs [34–38], and acting as metalloestrogens to promote breast tumor development [39–42]. Some studies reported higher arsenic and cadmium levels in breast cancer tissue than normal breast tissues [33, 43–46], and chromium was higher in colorectal tumor tissues than healthy tissues [47].

The epidemiologic literature on non-occupational exposure to arsenic, cadmium, chromium, or nickel in relation to breast cancer reports inconsistent results across ecological [48–50], case–control [51–61], case-cohort [62–64], and cohort studies [65–71]. To our knowledge, only ecological studies on heavy metals in relation to colorectal cancer rates have been published [48, 50]. Inconsistencies across the epidemiologic literature may be due in part to different assessments of heavy metal exposures in toenails [51, 52, 62], urine [53, 55, 56, 58–61, 63, 64], blood [54, 57, 69, 70], and area-based estimates of ambient concentrations [67, 68, 71] or water supply levels [65] that capture distinct time windows of exposure. In three large prospective cohort studies, higher estimates of ambient arsenic or cadmium and arsenic in water supply from the early 2000s were associated with higher risk of breast cancer [65, 68, 71]—suggesting that ambient levels may better capture longer-term exposures most relevant to carcinogenesis.

State tumor registries offer a unique advantage to study cancer incidence in large populations[72], particularly for communities that are underrepresented in epidemiologic studies of cancer [73] and disproportionately burdened by environmental contaminants. Kentucky (KY) men and women had the highest overall cancer rates in the US, and the second and third highest colorectal cancer rates in the US for men and women, respectively [1]. We utilized the KY Cancer Registry database and identified KY census tracts that are areas with high incidence of two common cancers: breast cancer and colorectal cancer. We then investigated whether ambient concentrations of known heavy metal carcinogens (i.e., arsenic, cadmium, hexavalent chromium, and nickel) were associated with residing in these areas of high cancer incidence. This is a novel research question to identify the distinct environmental characteristics of everyone diagnosed in Kentucky with breast cancer or colorectal cancer as it relates to living in cancer hotspot areas. This study expands upon conventional ecological studies—that correlate area-based environmental exposures and cancer rates in counties or provinces—to examine smaller geographic units (i.e., census tracts) and utilizing more detailed information on the cancer cases’ geocoded addresses to understand *each* case’s estimated exposure as opposed to the county’s estimated exposure used in conventional ecological studies. Distinct from existing cohort studies that utilize area-based exposure estimates in relation to cancer development within a specific enrolled cohort, this is a population registry-based study that investigated individual cancer cases and whether each of their census-tract level environmental exposures was associated with living in an area with high cancer incidence. This novel approach using cancer registry may help us to understand area-based drivers of cancer incidence in communities across the state.

## Methods

### Study population

The Kentucky Cancer Registry (KCR) is a population-based cancer registry that captures all KY cancer diagnoses since 1995. KCR is a National Cancer Institute (NCI) Surveillance Epidemiology and End Results (SEER) program site since 2000. Each case’s residential address at diagnosis was assigned their census tract using the 2010 Census designation. Registry information also included individuals’ demographic, health, and diagnostic information.

For this study, we obtained information on all primary female breast cancer (*n* = 77,679) and primary colorectal cancer (*n* = 56,633) diagnoses in KY from 1995–2018. We excluded those missing census tract information and those without outcome data, leaving a total analytic sample of 77,637 breast cancer cases and 56,598 colorectal cancer cases. This study was reviewed by the Institutional Review Board and determined that it was exempt.

### Outcome: residence in a *cancer* hotspot census-tract

We determined whether the cancer cases’ residence at diagnosis was located within a census tract with high cancer incidence (“hotspot”). The primary outcomes of interest were: 1) whether females with breast cancer resided in an invasive (i.e., Stage 1–4) breast cancer hotspot, and 2) whether individuals with colorectal cancer resided in a male or female colorectal cancer hotspot. To identify a hotspot census tract, we first counted the total number of invasive breast cancer cases and colorectal cancer cases in each of the 1115 KY census tracts. We then calculated age-standardized rates of female invasive breast cancer at the census-tract level using the age-distribution of KY females from the 2018 American Community Survey (ACS). For colorectal cancer, we calculated sex-specific age-standardized rates using the sex- and age-distribution of KY from the 2018 ACS. Six census tracts were excluded because of a population size of zero in 2018, leaving 1109 census tracts in the analysis.

To identify census tract hotspots, ArcGIS software (ArcGIS v.10, ESRI; Redlands, CA, USA) was used to calculate the Getis-Ord Gi* spatial statistic for the standardized rates of invasive breast cancer and colorectal cancer. Previous studies utilized the Getis-Ord Gi* statistic to identify cancer hotspot counties [74–76]. In brief, the Getis-Ord Gi* statistic is a measure of spatial autocorrelation, or the tendency of values of neighboring spatial units (using a queen contiguity spatial weight) to be more similar than values that are further away [77, 78]. Census tracts with a Z-score of greater than 1.96 indicated a clustering of census tracts with higher cancer rates than the average at the 95% confidence level.

### Exposures: census-tract estimates of ambient concentrations of arsenic, cadmium, chromium, and nickel

Estimated ambient concentrations of hazardous air pollutants (HAPs) were assigned to each KY census tract from the US Environmental Protection Agency (EPA) National Air Toxics Assessment (NATA) 2014 database. The EPA assessed HAPs approximately every three years from 1996–2014. The ambient HAP concentrations are compiled in the National Emissions Inventory—an inventory of outdoor emission sources. The nationwide annual ambient outdoor concentrations are estimated for census tracts via an air quality model integrating information on emissions, meteorology, and other information to account for atmospheric dynamics [79]. In this study, the 2014 NATA data were used for estimates of ambient concentrations (ng/m^3^) of arsenic compounds, cadmium, hexavalent chromium, and nickel for KY census tracts using the 2010 census tract designation; the 2010 designation allowed for linkage to additional census tract data to use as covariates. The 2002 NATA dataset uses the 2000 census tract designation and was used to compare the relative ranking of heavy metals concentrations over time; however, we did not combine multiple years of HAP estimates per NATA recommendations because different methodological approaches were used across assessment years [80]. We assigned the individual KY cancer cases to their corresponding estimated 2014 heavy metal concentrations in the census tract associated with their residence at diagnosis.

### Covariates

Individual-level information for the cases was available from the KCR that included age at diagnosis, sex for colorectal cancer analyses (male or female), race (Black, Other Races, White, or missing), marital status (single, married, separated/divorced/widowed, or missing), tobacco use (never used tobacco products, ever used tobacco products or cigarette-pack years > 0, or missing), family history of breast or colorectal cancer (yes, no, or missing), insurance status (none, private, public, or missing), and parity (parous, non-parous, or missing), and menopausal status for breast cancer analyses (yes, no, or missing).

Individual-level data was not available on socioeconomic status (SES) or other cancer risk factors. We utilized the 2010–2014 ACS 5-year estimates (U.S. Census Bureau, [81]) and the 2018 CDC Population Level Analysis and Community EStimates (PLACES) [82] to provide census-tract level estimates (2010 census tract designation) for population density, neighborhood SES and lifestyle factors. Census-tract level SES factors included the percentage of adults who graduated high school or higher, median household income, percentage of population below the poverty line, and percentage of the adult population that were not in the labor force that have been used previously [83]. Lifestyle variables related to breast or colorectal cancer risk were obtained from the 2018 CDC PLACES and included the percentage of physically inactive adults (i.e., no leisure-time physical activity among adults), prevalence of up-to-date cancer screening (e.g., mammography use among women aged 50–74 years, and fecal occult blood test, sigmoidoscopy, or colonoscopy among adults aged 50–75 years), and the percentage of current adult cigarette smokers.

### Analyses

**Fig. 1:**
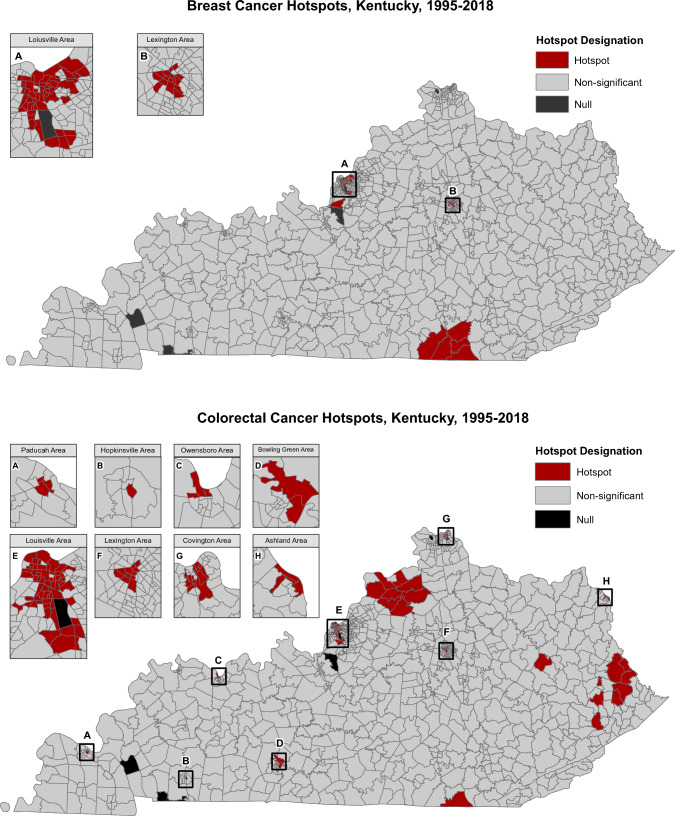
Distribution of Breast and Colorectal Hotspots Across 1,109 Kentucky Census Tracts. There are six “Null” census tracts in black that were excluded due to population sizes with less than one resident (ex. airports, army forts)

**Fig. 2:**
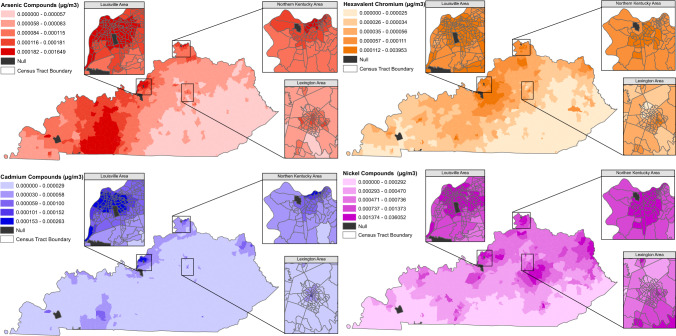
Distribution of Ambient Metals (μg/m^3^) Across Kentucky census tracts, 2014

ArcGIS was used to calculate the Getis-Ord Gi* statistic (see Outcome section above), to generate maps to visualize the hotspots (Fig. 1), and to create gradient maps showing the distribution of ambient heavy metal concentrations across KY census tracts (Fig. 2).

Due to the nature of population-based registries, we do not have residential histories prior to the person’s cancer diagnosis and cannot estimate long-term past exposure estimates. However, to understand the variability of potential exposure over a longer period of time, we calculated the Spearman correlation coefficient for each metal’s concentration in 2002 and 2014 to assess the relative ranking of each metal over twelve years (e.g., arsenic in 2002 and 2014) as other authors have suggested a minimum of ten years of residence exposure to air pollutants is necessary for tumor development [84]. For the main analyses, we used logistic regression models to estimate odds ratios (OR) and 95% confidence intervals (CI) for the association between each metal concentration and odds of residing in an invasive breast cancer hotspot (*n* = 77,637) or colorectal cancer hotspot (*n* = 56,598). The metal exposures were modeled continuously per a standard deviation (SD) increase and categorically using tertiles. Tests for trend across tertiles were calculated using the median value of each tertile level. We considered multiple models with adjustment variables and presented results from several models to show the magnitude of confounding. In brief, we first adjusted for individual-level characteristics that differed by hotspot status, then subsequently adjusted for population density and its quadratic term, followed by census-tract level characteristics that differed by hotspot status.

Breast cancer cases with missing values for median household income (*n* = 26), percentage of families below poverty line (*n* = 26), and mammography screening (*n* = 2) were assigned the median values. Similarly, colorectal cancer cases with missing values for census-tract median household income (*n* = 20), percentage of families below poverty line (*n* = 19), colorectal cancer screening (*n* = 3), percentage who smoked cigarettes (*n* = 1), and lack of physical activity (*n* = 1) were assigned the median values. A missing indicator variable was included in the model.

We conducted several sensitivity analyses. We excluded the extreme outliers [85] for ambient hexavalent chromium in breast cancer analyses (outliers *n* = 2718, 3.5%) and colorectal cancer analyses (outliers *n* = 2026, 3.6%). We examined whether the associations between metal concentrations and residence in a cancer hotspot were sensitive to urbanicity using the rural–urban commuting area (RUCA) classification C code [86, 87]. Because smoking is a major source of individual-level cadmium exposure [88], we ran the ambient cadmium analyses among never smokers to minimize potential residual confounding by smoking. Additionally, we ran a separate sensitivity analysis removing in situ Stage 0 breast cancer cases.

All regression analyses were conducted using SAS 9.4 (SAS Institute, Cary, NC, USA).

## Results

Of the 1,109 KY census tracts analysed, 73 were hotspots for invasive breast cancer (6.6%) and 115 were hotspots for male or female colorectal cancer (10.4%) (Fig. 1). The distributions of ambient arsenic, cadmium, hexavalent chromium, and nickel compounds across KY are shown in Fig. 2 and Supplemental Table 1, which are within the Occupational Safety and Health Administration (OSHA) standards for occupational levels [89, 90]. In general, carcinogenic metal concentrations were higher in KY urban centers than in more rural settings, due to many industries in KY located near urban areas, with an additional large area of high ambient arsenic in western KY (Fig. 2). The Spearman correlation coefficients assessing each metal concentration from 2002 and 2014 were 0.71 for arsenic, 0.85 for cadmium, 0.71 for chromium, and 0.70 for nickel (Supplemental Table 2) indicating high correlation over time that may reflect long-term exposure in non-movers.

**Table 1:** Individual and census-tract level characteristics of kentucky breast cancer cases (*n* = 77,637) and colorectal cancer cases (*n* = 56,598) by residence in a cancer hotspot

	Residence in a breast cancer hotspot (*n* = 5,044)	Residence was not in a breast cancer hotspot (*n* = 72,593)	Residence in a colorectal cancer hotspot (*n* = 5,290)	Residence was not in a colorectal cancer hotspot (*n* = 51,308)
Individual-level characteristics				
Age at diagnosis, mean (SD)	63.2 (14.0)	61.4 (13.5)	67.8 (13.3)	67.0 (13.6)
Male, % (n)	–	–	48.5 (2,564)	51.1 (26,204)
Race, % (n)				
Black	17.0 (855)	6.0 (4,348)	22.8 (1,208)	5.0 (2,561)
Other races	0.5 (25)	0.5 (380)	0.4 (23)	0.4 (198)
White	82.4 (4,156)	93.3 (67,692)	76.6 (4,051)	94.3 (48,366)
Missing	0.2 (8)	0.2 (173)	0.2 (8)	0.4 (183)
Marital status at diagnosis %(n)				
Single	14.6 (735)	7.1 (5,137)	13.8 (729)	7.3 (3,741)
Married	32.4 (1,635)	45.2 (32,814)	31.7 (1,679)	43.5 (22,329)
Separated/Divorced/Widowed	26.0 (1,310)	24.7 (17,894)	25.0 (1,320)	23.2 (11,898)
Missing	27.0 (1,364)	23.1 (16,748)	29.5 (1,562)	26.0 (13,340)
Parous, %(n)				
Yes	49.1 (2,476)	51.3 (37,214)	–	–
No	13.7 (692)	8.6 (6,271)	–	–
Missing	37.2 (1,876)	40.1 (29,108)	–	–
Tobacco use, %(n)				
Ever user	39.7 (2,001)	34.7 (25,221)	45.3 (2,396)	42.2 (21,635)
Never user	43.0 (2,171)	49.7 (36,075)	36.7 (1,942)	40.4 (20,707)
Missing/Unknown	17.3 (872)	15.6 (11,297)	18.0 (952)	17.5 (8,966)
Family History % (n)				
Yes	32.8 (1,654)	33.2 (24,104)	15.1 (801)	16.1 (8,261)
No	46.1 (2,324)	48.7 (35,331)	54.4 (2,877)	55.3 (28,349)
Missing	21.1 (1,066)	18.1 (13,158)	30.5 (1,612)	28.7 (14,698)
Insurance Status %(n)				
None	2.4 (119)	2.5 (1,789)	3.9 (204)	2.7 (1,373)
Private	29.8 (1,505)	30.0 (21,803)	16.0 (847)	18.5 (9,515)
Public	54.8 (2,762)	50.5 (36,641)	70.4 (3,726)	66.5 (34,094)
Unknown	13.1 (658)	17.0 (12,360)	9.7 (513)	12.3 (6,326)
Menopausal Status %(n)				
Pre-menopausal	17.7 (895)	21.1 (15,322)	–	–
Post-menopausal	69.0 (3,480)	66.5 (48,277)	–	–
Missing	13.3 (669)	12.4 (8,994)	–	–
Census-tract level characteristics				
Population density, people/sq. mi	4,366 (2,505–5,667)	359 (71–2,258)	3,696 (1,152–5,658)	225 (63–1,779)
% Families below the poverty line	16.9 (17.3)	12.9 (9.2)	23.6 (15.4)	13.4 (8.9)
Median Household Income, $	45,218 (26,668)	45,671 (19,196)	32,425 (13,077)	43,713 (17,861)
% Population with high school or greater	84.3 (11.5)	81.7 (10.1)	77.5 (8.8)	80.5 (10.2)
% Not in the labor force	37.7 (9.9)	39.0 (10.3)	41.6 (10.8)	40.2 (10.4)
% with up-to-date cancer screening	79.1 (2.2)	76.6 (2.3)	61.7 (4.7)	65.6 (4.7)
% Physically inactive	31.0 (9.6)	33.0 (6.7)	37.6 (6.9)	33.7 (6.5)
% Smoking	23.5 (9.3)	23.5 (5.2)	28.5 (6.2)	23.9 (5.1)
Ambient Arsenic, ng/m^3^	0.2206 (0.0935)	0.1229 (0.1022)	0.2048 (0.1000)	0.1182 (0.1039)
Ambient Cadmium, ng/m^3^	0.1184 (0.0668)	0.0415 (0.0414)	0.1117 (0.0738)	0.0381 (0.0383)
Ambient Hexavalent Chromium, ng/m^3^	0.2001 (0.1932)	0.0909 (0.1984)	0.1664 (0.1860)	0.0881 (0.2058)
Ambient Nickel, ng/m^3^	3.1892 (4.7844)	0.9982 (1.6583)	2.9437 (4.7894)	0.9295 (1.5299)

Values are means (SD) or medians (Q25, Q75) for continuous variables; percentages or ns or both for categorical variables

Values of categorical variables may not sum to 100% due to rounding

**Table 2:** The Odds Ratios (95% Confidence Intervals) of residing in a breast cancer hotspot (*n* = 77,637/events = 5,044) by tertiles of ambient Arsenic, Cadmium, Hexavalent Chromium, and Nickel and for a 1 Standard Deviation (SD) increase in each metal concentration (ng/m^3^)

	N/events	[min, max] or SD, ng/m^3^	Crude	Model 1^a^	Model 2^b^	Model 3^c^	Model 4^d^	Model 5^e^	Model 6^f^
Arsenic									
TERTILE 1	25,860/299	[0.0295–0.0781]	REF	REF	REF	REF	REF	REF	REF
TERTILE 2	25,856/1,152	[0.0782–0.1369]	3.98 (3.50, 4.53)	3.85 (3.38, 4.38)	1.62 (1.41, 1.85)	1.49 (1.30, 1.71)	1.63 (1.42, 1.87)	1.65 (1.44, 1.89)	2.78 (2.38, 3.23)
TERTILE 3	25,921/3,593	[0.13744–1.6491]	13.75 (12.20, 15.49)	12.40 (11.00, 13.99)	2.42 (2.12, 2.76)	2.33 (2.04, 2.65)	2.16 (1.89, 2.46)	2.09 (1.83, 2.38)	1.66 (1.43, 1.94)
*p*-trend			< 0.0001	< 0.0001	< 0.0001	< 0.0001	< 0.0001	< 0.0001	0.07
per SD of As	77,637/5,044	SD = 0.1044	1.77 (1.73, 1.82)	1.68 (1.63, 1.72)	1.43 (1.40, 1.46)	1.41 (1.38, 1.44)	1.36 (1.33, 1.39)	1.36 (1.33, 1.39)	1.36 (1.32, 1.39)
Cadmium									
TERTILE 1	25,909/299	[0.0075–0.0199]	REF	REF	REF	REF	REF	REF	REF
TERTILE 2	25,836/610	[0.0200–0.0377]	2.07 (1.80, 2.38)	1.99 (1.73, 2.29)	0.98 (0.85, 1.14)	0.86 (0.75, 1.00)	0.91 (0.78, 1.05)	0.91 (0.78, 1.05)	1.35 (1.15, 1.58)
TERTILE 3	25,892/4,135	[0.0380–0.2634]	16.28 (14.46, 18.33)	14.91 (13.23, 16.80)	3.14 (2.75, 3.59)	2.80 (2.46, 3.19)	2.75 (2.41, 3.15)	2.74 (2.40, 3.14)	2.18 (1.88, 2.54)
*p*-trend			< 0.0001	< 0.0001	< 0.0001	< 0.0001	< 0.0001	< 0.0001	< 0.0001
Per SD of Cd	77,637/5,044	SD = 0.0475	2.65 (2.60, 2.71)	2.71 (2.65, 2.78)	1.88 (1.83, 1.94)	1.81 (1.76, 1.86)	1.74 (1.69, 1.79)	1.73 (1.68, 1.79)	1.60 (1.55, 1.66)
Chromium(VI)									
TERTILE 1	25,853/664	[0.0049–0.0330]	REF	REF	REF	REF	REF	REF	REF
TERTILE 2	25,885/755	[0.0331–0.0799]	1.14 (1.03, 1.27)	1.15 (1.03, 1.28)	0.52 (0.46, 0.58)	0.46 (0.41, 0.51)	0.57 (0.50, 0.64)	0.53 (0.47, 0.60)	0.52 (0.46, 0.60)
TERTILE 3	25,899/3,625	[0.0802–3.9526]	6.17 (5.67, 6.72)	5.77 (5.30, 6.28)	1.28 (1.16, 1.41)	1.30 (1.18, 1.43)	1.39 (1.26, 1.54)	1.27 (1.15, 1.41)	0.89 (0.79, 1.01)
*p*-trend			< 0.0001	< 0.0001	< 0.0001	< 0.0001	< 0.0001	< 0.0001	< 0.0001
Per SD of Cr	77,637/5,044	SD = 0.1999	1.29 (1.27, 1.31)	1.24 (1.22, 1.26)	1.14 (1.12, 1.16)	1.11 (1.09, 1.14)	1.07 (1.05, 1.09)	1.07 (1.05, 1.10)	1.03 (1.00, 1.06)
Per SD of Cr*	74,919/4,439	SD = 0.0589	2.47 (2.41, 2.53)	2.44 (2.38, 2.50)	1.81 (1.76, 1.86)	1.84 (1.79, 1.90)	1.81 (1.76, 1.86)	1.81 (1.76, 1.86)	1.69 (1.63, 1.74)
Nickel									
TERTILE 1	25,865/299	[0.0749–0.4521]	REF	REF	REF	REF	REF	REF	REF
TERTILE 2	25,921/1,159	[0.4533–0.9263]	4.00 (3.52, 4.55)	4.02 (3.53, 4.57)	1.53 (1.33, 1.75)	1.39 (1.21, 1.60)	1.71 (1.49, 1.97)	1.66 (1.44, 1.91)	1.87 (1.61, 2.16)
TERTILE 3	25,851/3,586	[0.9266–36.0517]	13.76 (12.21, 15.51)	12.89 (11.44, 14.53)	2.57 (2.25, 2.93)	2.68 (2.35, 3.05)	2.90 (2.54, 3.31)	2.78 (2.42, 3.19)	2.31 (1.99, 2.68)
*p*-trend			< 0.0001	< 0.0001	< 0.0001	< 0.0001	< 0.0001	< 0.0001	< 0.0001
Per SD of Ni	77,637/5,044	SD = 2.0856	1.65 (1.61, 1.69)	1.60 (1.57, 1.64)	1.36 (1.33, 1.39)	1.39 (1.35, 1.42)	1.34 (1.30, 1.37)	1.34 (1.31, 1.37)	1.30 (1.26, 1.33)

^a^Model 1 is adjusted for age at diagnosis (years), race (Black, Other, White, or missing), and tobacco use (ever, never, or missing)

^b^Model 2 is adjusted for age at diagnosis (years), race (Black, Other, White, or missing), tobacco use (ever, never, or missing), and population density (population/square mile)

^c^Model 3 is adjusted for age at diagnosis (years), race (Black, Other, White, or missing), tobacco use (ever, never, or missing), population density (population/square mile), and % of the census tract that was up-to-date on mammography screening

^d^Model 4 is adjusted for age at diagnosis (years), race (Black, Other, White, or missing), tobacco use (ever, never, or missing), population density (population/square mile), % of the census tract that was up-to-date on mammography screening, and % of census tract that currently smokes cigarettes

^e^Model 5 is adjusted for age at diagnosis (years), race (Black, Other, White, or missing), tobacco use (ever, never, or missing), population density (population/square mile), % of the census tract that was up-to-date on mammography screening, % of census tract that currently smokes cigarettes, and census tract median household income ($)

^f^Model 6 is adjusted for age at diagnosis (years), race (Black, Other, White, or missing), tobacco use (ever, never, or missing), population density (population/square mile), % of the census tract that was up-to-date on mammography screening, % of census tract that currently smokes cigarettes, census tract median household income ($), marital status (married, single, separated, or missing), parity (nulliparous, parous, or missing), health insurance status (public, private, none, or missing), family history of breast cancer (yes, no or missing), % population not in the labor force, % population that has graduated high school, and % population that is physically inactive

^*^Per SD of Cr after excluding 2,718 extreme outliers

### Population characteristics of cancer cases

Table 1 presents the characteristics of the 77,637 female breast cancer cases and the 56,598 colorectal cancer cases. In this study, 5044 (6.5%) breast cancer cases resided in an invasive breast cancer hotspot. Among all 2.3 million KY women, 5.8% resided in a breast cancer hotspot. Compared to cases who did not reside in a breast cancer hotspot, the breast cancer cases that resided in an invasive breast cancer hotspot were older, had a higher proportion of people who were Black, single, nulliparous, postmenopausal, past or current tobacco users, and publicly insured. For the census-tract level characteristics, those living in a breast cancer hotspot lived in more densely populated areas, areas with a higher percentage of families living below the poverty line, higher education, more screening, less physical inactivity, and higher average ambient heavy metals.

There were 5290 (9.4%) colorectal cancer cases that lived in a colorectal cancer hotspot. Among the 4.4 million people living in KY in 2018, 8.5% of Kentuckians lived in a colorectal cancer hotspot. Cases that lived in a colorectal cancer hotspot had a higher proportion of people who were female, Black, single, smokers, and have public health insurance compared to cases who did not reside in a colorectal cancer hotspot. For the census-tract level characteristics, cases living in a colorectal cancer hotspot lived in more densely populated census tracts with a higher percentage of families living below the poverty line, a lower median household income, a lower percent of the population with a high school degree or higher, and a higher percentage of the population not in the labor force compared to those who did not live in a hotspot (Table 1). In addition, the hotspot cases were more likely to live in census tracts with a lower percentage of the population screened for colorectal cancer, a higher percentage of smokers, a greater percentage of the population who were physically inactive, and higher average ambient heavy metals compared to cases not in a hotspot.

### Heavy-metal exposures and odds of residing in a *cancer* hotspot

Breast cancer cases living in census tracts with higher concentrations of ambient arsenic, cadmium, chromium, or nickel had higher odds of residing in a breast cancer hotspot after adjustment for individual-level confounders and census-tract level confounders (Table 2). Model 1 includes adjustment for individual-level age, race, and tobacco use showing weak confounding relative to the crude results. The subsequent inclusion of population density and a quadratic term for population density (Model 2), census-tract level mammography screening (Model 3), smoking (Model 4), median household income (Model 5), and other variables (Model 6) attenuated the results—most notably population density, census-tract mammography screening and smoking. Among the breast cancer cases, a one SD increase in census-tract level ambient cadmium exposure was associated with 1.60-times higher odds of residing in a breast cancer hotspot (OR = 1.60 95% CI 1.55, 1.66; Model 6) after full covariate adjustment. While the remaining results were positive associations between carcinogenic metal concentrations and residence in a breast cancer hotspot, these associations were not as strong in magnitude when assessing SD increases in arsenic (OR = 1.36 95% CI 1.32, 1.39), chromium (OR = 1.03 95% CI 1.00, 1.06) or nickel (OR = 1.30 95% CI 1.26, 1.33). After excluding outlying values of hexavalent chromium, hexavalent chromium exposure was more strongly associated with residing in a breast cancer hotspot (OR = 1.69 95% CI 1.63, 1.74). When the metal concentrations were modeled as tertiles, the breast cancer cases with the highest tertile of ambient cadmium had 2.18-times higher odds of residing in a breast cancer hotspot than those residing in the lowest tertile of ambient cadmium (Tertile 3 vs. Tertile 1: OR = 2.18 95% CI 1.88, 2.54, Model 6). These associations were similar for arsenic (Tertile 3 vs. Tertile 1: OR = 1.66 95% CI 1.43, 1.94) and nickel (Tertile 3 vs. Tertile 1: OR = 2.31 95% CI 1.99, 2.68). The results were slightly stronger when the population was restricted to only urban breast cancer cases (Supplemental Table 3). The results were similar when we restricted to never smokers (Supplemental Table 4) and to invasive breast cancer cases (Supplementary Table 5).

**Table 3:** The Odds Ratios (95% Confidence Intervals) of Residing in a Colorectal Cancer Hotspot (*n* = 56,598/Events = 5290) by Tertiles of Ambient Arsenic, Cadmium, Hexavalent Chromium, and Nickel and for a 1 Standard Deviation (SD) Increase in each metal concentration (ng/m^3^)

	*n*/events	[min, max] or SD, ng/m^3^	Crude	Model 1^a^	Model 2^b^	Model 3^c^	Model 4^d^	Model 5^e^	Model 6^f^
Arsenic									
TERTILE 1	18,874/627	[0.0295–0.0739]	REF	REF	REF	REF	REF	REF	REF
TERTILE 2	18,840/993	[0.0742–0.1304]	1.62 (1.46, 1.79)	1.49 (1.34, 1.65)	0.98 (0.88, 1.10)	1.45 (1.30, 1.63)	1.45 (1.30, 1.63)	1.88 (1.67, 2.12)	2.47 (2.18, 2.80)
TERTILE 3	18,884/3,670	[0.1306–1.6491]	7.02 (6.43, 7.66)	5.89 (5.39, 6.43)	2.08 (1.86, 2.31)	3.58 (3.19, 4.01)	3.60 (3.21, 4.04)	3.19 (2.82, 3.60)	4.02 (3.52, 4.58)
*p*-trend			< 0.0001	< 0.0001	< 0.0001	< 0.0001	< 0.0001	< 0.0001	< 0.0001
per SD of As	56,598/5,290	SD = 0.1065	1.82 (1.77,1.87)	1.65 (1.60, 1.70)	1.31 (1.28, 1.34)	1.30 (1.27, 1.33)	1.28 (1.25, 1.31)	1.25 (1.22, 1.28)	1.27 (1.24, 1.30)
Cadmium									
TERTILE 1	18,822/627	[0.0075–0.0191]	REF	REF	REF	REF	REF	REF	REF
TERTILE 2	18,908/804	[0.0192–0.0331]	1.29 (1.16, 1.43)	1.17 (1.05, 1.30)	0.90 (0.81, 1.01)	1.59 (1.42, 1.79)	1.55 (1.38, 1.74)	1.81 (1.61, 2.04)	2.32 (2.05, 2.64)
TERTILE 3	18,868/3,859	[0.0333–0.2634]	7.46 (6.84, 8.14)	6.29 (5.76, 6.88)	2.45 (2.19, 2.73)	5.65 (5.02, 6.35)	5.54 (4.92, 6.24)	4.85 (4.28, 5.49)	5.65 (4.96, 6.44)
*p*-trend			< 0.0001	< 0.0001	< 0.0001	< 0.0001	< 0.0001	< 0.0001	< 0.0001
Per SD of Cd	56,598/5,290	SD = 0.0479	2.68 (2.62,2.74)	2.55 (2.49, 2.61)	2.08 (2.02, 2.15)	2.07 (2.01, 2.14)	2.07 (2.00, 2.13)	2.07 (1.99, 2.15)	2.08 (2.00, 2.16)
Chromium(VI)									
TERTILE 1	18,829/945	[0.0049–0.0309]	REF	REF	REF	REF	REF	REF	REF
TERTILE 2	18,884/710	[0.0310–0.0744]	0.74 (0.67, 0.82)	0.73 (0.66, 0.80)	0.54 (0.48, 0.60)	1.04 (0.93, 1.16)	1.05 (0.95, 1.17)	1.08 (0.97, 1.21)	1.23 (1.10, 1.38)
TERTILE 3	18,885/3,635	[0.0746–3.9526]	4.51 (4.19, 4.86)	3.97 (3.68, 4.29)	1.52 (1.39, 1.67)	3.56 (3.22, 3.93)	3.55 (3.21, 3.93)	2.75 (2.47, 3.07)	2.96 (2.63, 3.32)
* p*-trend			< 0.0001	< 0.0001	< 0.0001	< 0.0001	< 0.0001	< 0.0001	< 0.0001
Per SD of Cr	56,598/5,290	SD = 0.2053	1.24 (1.22,1.26)	1.16 (1.14,1.18)	1.01 (0.98, 1.04)	0.96 (0.93, 0.99)	0.96 (0.93, 0.98)	0.90 (0.87, 0.93)	0.89 (0.86, 0.92)
Per SD of Cr*	54,572/4,759	SD = 0.0591	1.99 (1.95, 2.04)	1.91 (1.87, 1.96)	1.45 (1.40, 1.49)	1.59 (1.54, 1.64)	1.62 (1.57, 1.68)	1.51 (1.45, 1.56)	1.51 (1.45, 1.56)
Nickel									
TERTILE 1	18,849/479	[0.0749–0.3941]	REF	REF	REF	REF	REF	REF	REF
TERTILE 2	18,906/1,226	[0.3958–0.8688]	2.66 (2.39, 2.96)	2.61 (2.34, 2.90)	1.73 (1.55, 1.94)	2.81 (2.51, 3.15)	2.90 (2.59, 3.26)	2.80 (2.49, 3.15)	3.38 (3.00, 3.81)
TERTILE 3	18,843/3,585	[0.8715–36.0517]	9.01 (8.17, 9.94)	8.04 (7.28, 8.87)	3.04 (2.72, 3.41)	6.33 (5.62, 7.12)	6.51 (5.78, 7.32)	4.85 (4.27, 5.49)	5.58 (4.90, 6.36)
*p*-trend			< 0.0001	< 0.0001	< 0.0001	< 0.0001	< 0.0001	< 0.0001	< 0.0001
Per SD of Ni	56,598/5,290	SD = 2.1469	1.83 (1.78, 1.88)	1.72 (1.67, 1.77)	1.36 (1.32, 1.40)	1.28 (1.25, 1.32)	1.29 (1.25, 1.33)	1.19 (1.15, 1.23)	1.21 (1.17, 1.25)

^a^Model 1 is adjusted for age at diagnosis (years), race (Black, Other, White, or missing), sex (male, female), and tobacco use (ever, never, or missing)

^b^Model 2 is adjusted for age at diagnosis (years), race (Black, Other, White, or missing), sex (male, female), tobacco use (ever, never, or missing) and population density (population/square mile)

^c^Model 3 is adjusted for age at diagnosis (years), race (Black, Other, White, or missing), sex (male, female), tobacco use (ever, never, or missing), population density (population/square mile) and census tract median income ($)

^d^Model 4 is adjusted for age at diagnosis (years), race (Black, Other, White, or missing), sex (male, female), tobacco use (ever, never, or missing), population density (population/square mile), census tract median income ($) and % population physically inactive

^e^Model 5 is adjusted for age at diagnosis (years), race (Black, Other, White, or missing), sex (male, female), tobacco use (ever, never, or missing), population density (population/square mile), census tract median income ($), % population physically inactive, % of census tract that currently smokes cigarettes, and % of census tract that was up-to-date on colorectal cancer screening

^f^Model 6 is adjusted for age at diagnosis (years), race (Black, Other, White, or missing), sex (male, female), tobacco use (ever, never, or missing), population density (population/square mile), census tract median income ($), % population physically inactive, % of census tract that currently smokes cigarettes, % of census tract that was up-to-date on colorectal cancer screening, marital status (single, married, separated or divorced or widowed, and missing), family history of colorectal cancer (yes, no, or missing), health insurance (none, private, public, or missing), % of census tract who graduated high school, and % not in the labor force

^*^Per SD of Cr after excluding 2,026 extreme outliers

Among colorectal cancer cases, heavy metal exposures showed positive associations with residing in a colorectal cancer hotspot (Table 3). Among colorectal cancer cases, a one SD increase of ambient cadmium exposure was associated with 2.08-times higher odds of residing in a colorectal cancer hotspot in the fully adjusted model (OR = 2.08 95% CI 2.00, 2.16; Table 3, Model 6) that adjusted for age, race, sex, tobacco use, marital status, family history of colorectal cancer, health insurance, population density and its quadratic term, census-tract level colorectal cancer screening, census-tract physical inactivity, census-tract smoking, census-tract SES markers. Positive associations were also observed for a SD increase in ambient arsenic (OR = 1.27 95% CI 1.24, 1.30) and nickel (OR = 1.21 95% CI 1.17, 1.25). When excluding those with extreme outlying values of hexavalent chromium, ambient hexavalent chromium was associated with higher odds of residing in a colorectal cancer hotspot (OR = 1.51 95% CI 1.45, 1.56). The results were slightly stronger when the population was restricted to urban colorectal cancer cases (Supplementary Table 6). The results were similar for never smokers (Supplementary Table 7).

In brief, breast and colorectal cancer cases living in census tracts with higher concentrations of ambient arsenic, cadmium, hexavalent chromium, or nickel had higher odds of residing in a breast or colorectal cancer hotspot after adjustment for individual-level confounders and census-tract level confounders (Table 2). After full covariate adjustment, people with breast cancer who lived in census tracts with a one SD higher exposure to ambient cadmium, ambient arsenic, and ambient nickel had 1.60 (95% CI 1.55, 1.66; Model 6), 1.36 (95% CI 1.32, 1.39), and 1.30 (95% CI 1.26, 1.33) times higher odds of residing in a breast cancer hotspot. After full covariate adjustment, people with colorectal cancer who lived in census tracts with a one SD higher exposure to ambient cadmium, ambient arsenic, and ambient nickel had 2.08 (95% CI 2.00, 2.16; Model 6), 1.27 (95% CI 1.24, 1.30), and 1.21 (95% CI 1.17, 1.25) times higher odds of residing in a colorectal cancer hotspot.

## Discussion

In summary, we observed that cancer cases who lived in census tracts with higher exposures to ambient heavy metal carcinogens had higher odds of living in colorectal and breast cancer hotspots within Kentucky, independent of individual-level risk factors and census-tract level risk factors. The results were most striking for ambient cadmium exposure in relation to both breast cancer and colorectal cancer hotspots, and they were slightly stronger when restricted to cases who lived in urban areas. Additionally, the cases that resided in a cancer hotspot were more likely to be Black, have public health insurance, living in low-income areas, and smokers (Table 1), which are characteristics of the populations facing much of the burden of these environmental exposures that influence cancer risk—as reported in other US areas [25, 29, 91, 92].

Stark differences were seen in the characteristics of cases who lived in hotspots versus cases not living in hotspots in terms of race, poverty, marital status, health insurance, and population density. Similar to our study, Moore et al*.* identified breast cancer mortality hotspot counties that were located in the Southern US and had residents who were more likely to be Black or lower income, among other characteristics [74]. Additionally, survival after an early onset colorectal cancer diagnosis was worse for those who resided in hotspot counties found in the South and were non-Hispanic Black men [93]. In the current study, people living in areas with higher ambient carcinogenic heavy metal concentrations had higher odds of living in cancer hotspots. These results are similar to other studies that showed a higher burden of cancer and ambient metal contamination in communities with higher poverty, higher Black populations or more racial segregation [25, 29, 91, 92]. In a study of urban soil samples in eight southern US cities (including two KY cities), soil cadmium concentrations were higher in more impoverished areas and areas with a higher percentage of the population who were racial minorities; soil arsenic was also higher in predominately non-White communities [26]. The findings in our study provide additional evidence on the populations facing much of the burden of these environmental exposures that contribute to cancer risk within KY, specifically people who are Black living in poor urban communities.

Most studies on metals and breast cancer have focused on heavy metal concentrations in toenails [51, 52, 62], urine [53, 55, 56, 58–61, 63, 64], and blood [54, 57, 69, 70], whereas a limited number have investigated ambient concentrations [67, 68, 71]. In the California Teachers Study (CTS), Liu et al*.* examined eleven estrogen-disrupting HAPs, including arsenic and cadmium compounds, among 112,379 females of whom 5,361 developed breast cancer. They observed that higher census-tract level exposures to ambient cadmium and arsenic were associated with higher rates of hormone receptor-negative breast cancer [68], that disproportionately affects non-Hispanic Black women. Additionally, in the nationwide US Sister Study Cohort, higher ambient cadmium had a suggestive association with higher breast cancer incidence in postmenopausal women [71]. However, in the Nurses’ Health Study II (NHSII), Hart et al*.* examined a nationwide cohort of women to assess the impact of HAPs and breast cancer incidence, and did not observe an association between ambient arsenic exposure and rates of invasive breast cancer [67]. Key differences in the cohorts may explain the inconsistent findings. The CTS cohort was mostly post-menopausal, nulliparous, and had a higher proportion of non-White populations compared to NHSII [68]. In our study, we found that the breast cancer cases that resided in hotspots had a higher prevalence of being Black, postmenopausal, and nulliparous that is more similar to the CTS study. This similarity suggests that these ambient heavy metal exposures, especially arsenic and cadmium, may contribute as risk factors for invasive breast cancer in non-White, postmenopausal and nulliparous women and should be investigated in future studies.

Cadmium had the strongest association with residence in KY cancer hotspots. Stronger associations between cadmium and cancer hotspots may be due to its long half-life and its ability to bioaccumulate [94]. Of the listed metals, cadmium has the longest half-life of 4 to 38 years in bodily organs, with a slower rate of renal excretion compared to the other heavy metals [95]. Additionally, there are high amounts of cadmium in tobacco leaves, cigarettes, and cigarette smoke [88]. In our study, even after adjusting for individual’s tobacco use and restricting to never smokers, higher ambient cadmium exposure was still associated with higher risk of residing in a hotspot. This higher risk suggests that ambient sources of cadmium, irrespective of cigarette smoking, may play a role in breast cancer and colorectal cancer incidence.

Arsenic, cadmium, hexavalent chromium and nickel are characterized as potent endocrine disruptors and metalloestrogens, which are known to bind to estrogen receptors (ER) and affect estrogen regulated genes, as well as induce proliferation of estrogen-dependent cancer cells, aberrant cellular proliferation, and breast tumorigenesis [41, 42, 96, 97]. At subtoxic levels, cadmium in MCF-7 breast cancer cells impaired TP53 function [98]. Short-term arsenic exposure activates ER signaling, while chronic exposure suppresses ER expression and signaling [96]. Additionally, chronic arsenic exposure induced carcinogenesis in healthy human breast epithelial cells through overexpression of aromatase [99]. Danes et al*.* observed that arsenic promotes ER-positive epithelial breast cancer cells to transition to a basal phenotype, with increased proliferation and more aggressive phenotypes with greater metastatic potential in vivo [96].

While other environmental factors are hypothesized to contribute to colorectal cancer development, to our knowledge, there has not been an individual-level epidemiologic study on heavy metal exposures and colorectal cancer incidence. In ecologic studies in Iran, Spain, and Argentina, higher arsenic and cadmium concentrations in soil or groundwater were correlated with higher rates of colorectal cancer incidence or mortality [11, 48, 50]. Sohrabi et al*.* reported increased levels of chromium in colorectal tumor tissue specimens compared to healthy tissues [47]. Cadmium induced migration of colorectal cancer cells through activation of key pathways that promote cell proliferation, survival, invasion, and metastasis [34]. Our study suggests that living in areas with higher ambient heavy metal concentrations was associated with living in a colorectal cancer hotspot, and suggests that additional individual-level epidemiologic studies are needed to examine these metals as potential risk factors for colorectal cancer.

Our study has several limitations. We used the 2014 NATA HAPs estimates of ambient exposures for cancer cases diagnosed between 1995 and2018. Due to the nature of population-based registries, we do not have residential histories prior to the person’s cancer diagnosis and cannot estimate long-term past exposure estimates, though an estimated 85% of Kentuckians stay in the same residence over 1 year [100]. The lack of temporality between the true metal exposures and developing cancer and the unknown duration of exposure in this study precludes any causal conclusions regarding these metals’ role on tumor initiation. However, we reported high correlations between the 2002 and 2014 ambient metal concentrations in KY, which indicates that the relative ranking of ambient concentrations was stable over a twelve year period. Therefore, these findings may reflect the minimum time of residential exposure necessary for carcinogenesis [84] for non-movers in Kentucky. Additionally, ecological estimates of ambient metal concentrations of the residence at diagnosis were used, which may not capture true personal level exposure, residential histories or cumulative metal exposures prior to diagnosis. However, large prospective cohort studies also utilized the NATA databases for geographical estimates of exposure and reported intriguing results regarding breast cancer risk [67, 68, 71]. Additionally, we were not able to adjust for all known individual-level cancer risk factors (e.g., obesity, dietary information such as alcohol for both cancers and processed or red meats for CRC) in this population-based study that could lead to residual confounding if related to ambient heavy metal exposure; however, individual-level risk factors were weaker confounders than the census-tract characteristics in the statistical models. We also could not account for changes in residence prior to diagnosis or occupational exposures to metal carcinogens. Despite these limitations, this work contributes to the limited body of literature on ambient metal exposures influencing breast and colorectal cancer risk at the community-level in the general population and was a novel approach within population-based registries.

In conclusion, we observed that among KY cancer cases, living in areas with higher ambient concentrations of cadmium, arsenic, hexavalent chromium, and nickel was associated with living in breast and colorectal cancer hotspots in KY, independent of individual and neighborhood risk factors. Additionally, we observed that those living in cancer hotspot communities were more likely to be Black and living in poverty (Table 1), and existing literature has reported that these populations also have higher exposure to ambient metal pollutants [25–31], suggesting that structural racism and environmental injustice contribute to cancer development in communities. Linking administrative cancer surveillance data with publicly available environmental exposures allows for research to examine potential harms more efficiently for large populations. In addition, these large databases allow estimation of potential harms within underrepresented or marginalized groups, offering opportunities to identify modifiable contributors to health disparities. Our findings suggest that exposures to these non-essential heavy metals at ambient concentrations present in the hotspots may be potential risk factors for breast and colorectal cancer worthy of further investigation in individual-level epidemiologic studies.

## Supplementary Information

Below is the link to the electronic supplementary material.

Supplementary file1 (DOCX 28 kb)

## Data Availability

The 2014 EPA NATA dataset used in this study is publicly available from the US EPA NATA 2014 Assessment Results: https://www.epa.gov/national-air-toxics-assessment/2014-nata-assessment-results. The US Census Bureau's American Community Survey datasets are publicly available online: https://www.census.gov/programs-surveys/acs/data.html. The Kentucky Cancer Registry datasets analyzed in the current study are not publicly available due to the data containing protected health information. The data can be requested from the Kentucky Cancer Registry (https://www.kcr.uky.edu/research/) with appropriate IRB approvals.
