# Supplementary material for: Heavy-metal associated breast cancer and colorectal cancer hot spots and their demographic and socioeconomic characteristics
Source: Cancer Causes Control. 2024 Jun 25;35(10):1367–81. doi: 10.1007/s10552-024-01894-0 (PMC11461597; doi:10.1007/s10552-024-01894-0)
Supplement: Supplementary file 1 — Supplementary file1 (DOCX 28 kb) [file 10552_2024_1894_MOESM1_ESM.docx]

| **Supplemental Table 1.** Distribution of Census-Tract Level Metal Concentrations (ng/m^3^) in Kentucky census tracts (n=1,109) | | | |
| --- | --- | --- | --- |
|  | Mean (SD) | Min-Max | Median [IQR] |
| Arsenic | 0.1268 (0.1035) | 0.0295-1.6491 | 0.0976 [0.0626-0.1616] |
| Cadmium | 0.0456 (0.0489) | 0.0075-0.2634 | 0.0244 [0.0172-0.0510] |
| Hexavalent Chromium | 0.0935 (0.2047) | 0.0049-3.9526 | 0.0424 [0.0273-0.0977] |
| Nickel | 1.1453 (2.1793) | 0.0749-36.0517 | 0.5820 [0.3330-1.1318] |

**Supplemental Table 2**. Spearman Correlation matrix of ambient metals from 2014 and 2002 US EPA NATA assessment years

|  | 2002 Arsenic | 2014 Arsenic | 2002 Cd | 2014 Cd | 2002 Cr(VI) | 2014 Cr(VI) | 2002 Ni | 2014 Ni |
| --- | --- | --- | --- | --- | --- | --- | --- | --- |
| 2002 As | 1 |  |  |  |  |  |  |  |
| 2014 As | 0.71 | 1 |  |  |  |  |  |  |
| 2002 Cd | 0.80 | 0.75 | 1 |  |  |  |  |  |
| 2014 Cd | 0.78 | 0.87 | 0.85 | 1 |  |  |  |  |
| 2002 Cr(VI) | 0.81 | 0.70 | 0.72 | 0.74 | 1 |  |  |  |
| 2014 Cr(VI) | 0.67 | 0.73 | 0.73 | 0.82 | 0.71 | 1 |  |  |
| 2002 Ni | 0.82 | 0.76 | 0.80 | 0.86 | 0.87 | 0.87 | 1 |  |
| 2014 Ni | 0.67 | 0.56 | 0.69 | 0.76 | 0.66 | 0.66 | 0.70 | 1 |

**Supplemental Table 3**. Among the *Urban-Only breast cancer residents (n=43,683/events=4,776)*, the Odds Ratios (95% Confidence Intervals) of Residing in a Breast Cancer Hotspot by Tertiles of Ambient Arsenic, Cadmium, Hexavalent Chromium, and Nickel and for a 1 Standard Deviation (SD) Increase in each metal (ng/m^3^).

|  | **N/Events** | **[min, max] or SD, ng/m^3^** | **Crude** | **Model 1^a^** | **Model 2^b^** |
| --- | --- | --- | --- | --- | --- |
| **Arsenic** |  |  |  |  |  |
| TERTILE 1 | 14,552/ 535 | [0.0364-0.1097] | REFERENT | REFERENT | REFERENT |
| TERTILE 2 | 14,622/ 823 | [0.1101-0.1781] | 1.56 (1.40, 1.75) | 1.21 (1.08, 1.36) | 0.83 (0.73, 0.94) |
| TERTILE 3 | 14,509/ 3,418 | [0.1782-0.6805] | 8.07 (7.35, 8.87) | 3.60 (3.24, 3.98) | 2.16 (1.92, 2.44) |
| per SD of As | 43,683/ 4,776 | SD= 0.0780 | 2.35 (2.28, 2.42) | 1.96 (1.89, 2.03) | 1.80 (1.73, 1.88) |
| **Cadmium** |  |  |  |  |  |
| TERTILE 1 | 14,580/ 345 | [0.0096-0.0306] | REFERENT | REFERENT | REFERENT |
| TERTILE 2 | 14,542/ 981 | [0.0307-0.0745] | 2.98 (2.63, 3.38) | 3.22 (2.82, 3.66) | 2.09 (1.83, 2.39) |
| TERTILE 3 | 14,561/ 3,450 | [0.0746-0.2634] | 12.81 (11.44, 14.35) | 4.95 (4.38, 5.58) | 2.69 (2.35, 3.08) |
| per SD of Cd | 43,683/ 4,776 | SD= 0.0541 | 2.55 (2.48, 2.62) | 1.89 (1.82, 1.96) | 1.76 (1.69, 1.84) |
| **Chromium(VI)** |  |  |  |  |  |
| TERTILE 1 | 14,575/ 932 | [0.0157-0.0604] | REFERENT | REFERENT | REFERENT |
| TERTILE 2 | 14,565/ 260 | [0.0606-0.1185] | 0.27 (0.23, 0.31) | 0.24 (0.21, 0.28) | 0.19 (0.16, 0.22) |
| TERTILE 3 | 14,543/ 3,584 | [0.1191-2.3013] | 4.79 (4.44, 5.17) | 2.60 (2.39, 2.83) | 1.87 (1.69, 2.07) |
| Per SD of Cr | 43,683/ 4,776 | SD=0.1971 | 1.30 (1.28, 1.33) | 1.08 (1.05, 1.11) | 1.03 (1.00, 1.06) |
| Per SD of Cr* | 41,275/ 4,171 | SD=0.0607 | 2.35 (2.29, 2.42) | 1.98 (1.91, 2.04) | 1.88 (1.81, 1.95) |
| **Nickel** |  |  |  |  |  |
| TERTILE 1 | 14,584/ 779 | [0.1345-0.7528] | REFERENT | REFERENT | REFERENT |
| TERTILE 2 | 14,508/ 1,011 | [0.7572-1.3416] | 1.33 (1.21, 1.46) | 0.80 (0.72, 0.89) | 0.68 (0.60, 0.76) |
| TERTILE 3 | 14,591/ 2,986 | [1.3570-36.0517] | 4.56 (4.20, 4.95) | 2.03 (1.85, 2.24) | 1.44 (1.29, 1.61) |
| Per SD of Ni | 43,683/ 4,776 | SD=2.3084 | 2.17 (2.09, 2.26) | 1.83 (1.74, 1.92) | 1.61 (1.53, 1.69) |

^a^Model 1 is adjusted for age at diagnosis (years), race (Black, Other, White, or missing), tobacco use (ever, never, or missing), population density (population/square mile), % of the census tract that was up-to-date on mammography screening, % of census tract that currently smokes cigarettes, and census tract median household income ($).

^b^Model 2 is adjusted for age at diagnosis (years), race (Black, Other, White, or missing), tobacco use (ever, never, or missing), population density (population/square mile), % of the census tract that was up-to-date on mammography screening, % of census tract that currently smokes cigarettes, census tract median household income ($), marital status (married, single, separated, or missing), parity (nulliparous, parous, or missing), health insurance status (public, private, none, or missing), family history of breast cancer (yes, no or missing), % population not in the labor force, % population that has graduated high school, and % population that is physically inactive

*Per SD of Cr after excluding extreme outliers

**Supplemental Table 4.** Among the Never-Smoking breast cancer cases (n=38,246/events=2,171), the Odds Ratios (95% Confidence Intervals) of Residing in a Breast Cancer Hotspot by Tertiles of Ambient Cadmium and for a 1 Standard Deviation (SD) Increase in Ambient Cadmium (ng/m^3^).

|  | **N/ Events** | **[min, max] or SD, ng/m^3^** | **Crude** | **Model 1^a^** | **Model 2^b^** |
| --- | --- | --- | --- | --- | --- |
| **Cadmium** |  |  |  |  |  |
| TERTILE 1 | 12,748/ 124 | [0.0075-0.0197] | REFERENT | REFERENT | REFERENT |
| TERTILE 2 | 12,753/ 292 | [0.0198-0.0349] | 2.39 (1.93, 2.95) | 1.05 (0.84, 1.31) | 1.57 (1.24, 1.98) |
| TERTILE 3 | 12,745/ 1,755 | [0.0350-0.2634] | 16.26 (13.53, 19.54) | 3.05 (2.49, 3.73) | 2.44 (1.95, 3.06) |
| per SD of Cd | 38,246/ 2,171 | SD= 0.0444 | 2.53 (2.45, 2.61) | 1.71 (1.64, 1.78) | 1.57 (1.49, 1.65) |

^a^Model 1 is adjusted for age at diagnosis (years), race (Black, Other, White or missing), population density, % of the census tract that has been screened for breast cancer, % of census tract that currently smokes cigarettes, and census tract median household income ($).

^b^Model 2 is adjusted for age at diagnosis (years), race (Black, Other, White or missing), marital status (single, married, separated, or missing), parous (yes, no, or missing), insurance status (private, public, or missing), family history of breast cancer (yes, no or missing), population density, % of the census tract that has been screened for breast cancer, % of census tract that currently smokes cigarettes, census tract median household income ($), % of population not in the labor force, % population graduated high school, and % population physically inactive.

**Supplemental Table 5**. Among invasive (Stage 1-4) breast cancer cases (n=62,082/events=4,069), the Odds Ratios (95% Confidence Intervals) of Residing in a Breast Cancer Hotspot for a 1 Standard Deviation (SD) Increase in Ambient Arsenic, Cadmium, Hexavalent Chromium, and Nickel (ng/m^3^).

|  | **N/ Events** | **SD, ng/m^3^** | **Crude** | **Model 1^a^** | **Model 2^b^** |
| --- | --- | --- | --- | --- | --- |
| **Arsenic** |  |  |  |  |  |
| per SD of As | 62,082/ 4,069 | SD= 0.1029 | 1.85 (1.79, 1.91) | 1.36 (1.33, 1.40) | 1.36 (1.32, 1.40) |
| **Cadmium** |  |  |  |  |  |
| per SD of Cd | 62,082/ 4,069 | SD= 0.0476 | 2.67 (2.61, 2.74) | 1.74 (1.68, 1.80) | 1.61 (1.55, 1.67) |
| **Chromium(VI)** |  |  |  |  |  |
| Per SD of Cr | 62,082/ 4,069 | SD=0.2000 | 1.29 (1.27, 1.32) | 1.07 (1.05, 1.10) | 1.04 (1.01, 1.07) |
| Per SD of Cr* | 59,898/ 3,572 | SD=0.0591 | 2.47 (2.40, 2.53) | 1.80 (1.74, 1.86) | 1.68 (1.62, 1.74) |
| **Nickel** |  |  |  |  |  |
| Per SD of Ni | 62,082/ 4,069 | SD=2.1063 | 1.64 (1.60, 1.68) | 1.32 (1.29, 1.36) | 1.29 (1.25, 1.32) |

^a^Model 1 is adjusted for age at diagnosis (years), race (Black, Other, White, or missing), tobacco use (ever, never, or missing), population density (population/square mile), % of the census tract that has been screened for breast cancer, % of census tract that currently smokes cigarettes, and census tract median household income ($).

^b^Model 2 is adjusted for age at diagnosis (years), race (Black, Other, White, or missing), tobacco use (ever, never, or missing), marital status (married, single, separated, or missing), insurance status (public, private, none, or missing), family history (yes, no or missing), population density (population/square mile), % of the census tract that has been screened for breast cancer, % of census tract that currently smokes cigarettes, census tract median household income ($), % population not in the labor force, % population that has graduated high school, and % population that is physically inactive.

*Per SD of Cr after excluding extreme outliers.

**Supplemental Table 6**. Among the *Urban-Only colorectal cancer residents (n=29,117/events=4,346)*, the Odds Ratios (95% Confidence Intervals) of Residing in a Colorectal Cancer Hotspot by Tertiles of Ambient Arsenic, Cadmium, Hexavalent Chromium, and Nickel and for a 1 Standard Deviation (SD) Increase in each metal (ng/m^3^).

|  | **N/Events** | **[min, max] or SD, ng/m^3^** | **Crude** | **Model 1^a^** | **Model 2^b^** |
| --- | --- | --- | --- | --- | --- |
| **Arsenic** |  |  |  |  |  |
| TERTILE 1 | 9,728/ 460 | [0.0364-0.1091] | REFERENT | REFERENT | REFERENT |
| TERTILE 2 | 9,716/ 951 | [0.1091-0.1827] | 2.19 (1.95, 2.45) | 1.89 (1.66, 2.16) | 1.79 (1.57, 2.05) |
| TERTILE 3 | 9,673/ 2,935 | [0.1837-0.6805] | 8.78 (7.92, 9.73) | 2.57 (2.25, 2.93) | 2.34 (2.05, 2.68) |
| per SD of As | 29,117/ 4,346 | SD=0.0835 | 2.64 (2.55, 2.73) | 1.67 (1.61, 1.74) | 1.69 (1.62, 1.76) |
| **Cadmium** |  |  |  |  |  |
| TERTILE 1 | 9,704/ 407 | [0.0096-0.0303] | REFERENT | REFERENT | REFERENT |
| TERTILE 2 | 9,732/ 695 | [0.0306-0.0752] | 1.76 (1.55, 1.99) | 1.93 (1.68, 2.21) | 1.84 (1.60, 2.11) |
| TERTILE 3 | 9,681/ 3,244 | [0.0754-0.2634] | 11.51 (10.34, 12.82) | 7.92 (6.81, 9.20) | 6.94 (5.96, 8.08) |
| per SD of Cd | 29,117/ 4,346 | SD=0.0561 | 3.11 (3.01, 3.21) | 2.45 (2.33, 2.57) | 2.40 (2.28, 2.53) |
| **Chromium(VI)** |  |  |  |  |  |
| TERTILE 1 | 9,758/ 612 | [0.0157-0.0601] | REFERENT | REFERENT | REFERENT |
| TERTILE 2 | 9,672/ 1,155 | [0.0604-0.1200] | 2.03 (1.83, 2.24) | 2.15 (1.90, 2.43) | 2.00 (1.77, 2.27) |
| TERTILE 3 | 9,687/ 2,579 | [0.1200-2.3013] | 5.42 (4.94, 5.95) | 2.96 (2.61, 3.36) | 2.68 (2.35, 3.05) |
| Per SD of Cr | 29,117/ 4,346 | SD=0.2062 | 1.26 (1.23, 1.30) | 0.86 (0.83, 0.89) | 0.83 (0.80, 0.86) |
| Per SD of Cr* | 27,316/ 3,815 | SD=0.0626 | 2.02 (1.95, 2.08) | 1.63 (1.56, 1.70) | 1.57 (1.50, 1.64) |
| **Nickel** |  |  |  |  |  |
| TERTILE 1 | 9,714/ 799 | [0.1345-0.7451] | REFERENT | REFERENT | REFERENT |
| TERTILE 2 | 9,715/ 653 | [0.7469-1.3941] | 0.80 (0.72, 0.90) | 0.99 (0.87, 1.14) | 1.00 (0.87, 1.15) |
| TERTILE 3 | 9,688/ 2,894 | [1.3965-36.0517] | 4.75 (4.37, 5.17) | 1.97 (1.75, 2.22) | 1.78 (1.57, 2.01) |
| Per SD of Ni | 29,117/ 4,346 | SD=2.5248 | 3.28 (3.10, 3.48) | 1.33 (1.26, 1.39) | 1.28 (1.22, 1.35) |

*Model 1 is adjusted for age at diagnosis (years), race (Black, Other, White, or missing), sex (male or female), tobacco use (ever, never, or missing), population density (population/square mile), census tract median income ($), % population physically inactive, % of census tract that currently smokes cigarettes, and % of census tract that was up-to-date on colorectal cancer screening.

**Model 2 is adjusted for age at diagnosis (years), race (Black, Other, White, or missing), sex (male, female), tobacco use (ever, never, or missing), population density (population/square mile), census tract median income ($), % population physically inactive, % of census tract that currently smokes cigarettes, % of census tract that was up-to-date on colorectal cancer screening, marital status (single, married, separated or divorced or widowed, and missing), family history of colorectal cancer (yes, no, or missing), health insurance (none, private, public, or missing), % of census tract who graduated high school, and % not in the labor force.

*Per SD of Cr after excluding extreme outliers.

**Supplemental Table 7.** Among the Never-Smoking colorectal cancer cases (n=22,649/events=1,942), the Odds Ratios (95% Confidence Intervals) of Residing in a Colorectal Cancer Hotspot by Tertiles of Ambient Cadmium and for a 1 Standard Deviation (SD) Increase in Ambient Cadmium (ng/m^3^).

| **Cadmium** | **N/ Events** | **[min, max] or SD, ng/m^3^** | **Crude** | **Model 1^a^** | **Model 2^b^** |
| --- | --- | --- | --- | --- | --- |
| TERTILE 1 | 7,542/ 283 | [0.0075-0.0189] | REFERENT | REFERENT | REFERENT |
| TERTILE 2 | 7,543/ 273 | [0.0190-0.0317] | 0.96 (0.81, 1.14) | 1.38 (1.15, 1.67) | 1.87 (1.53, 2.29) |
| TERTILE 3 | 7,564/ 1,386 | [0.0318-0.2634] | 5.75 (5.04, 6.57) | 4.17 (3.45, 5.06) | 5.14 (4.19, 6.31) |
| per SD of Cd | 22,649/ 1,942 | SD=0.0452 | 2.48 (2.39, 2.57) | 2.05 (1.93, 2.17) | 2.07 (1.95, 2.20) |

*Model 1 is adjusted for age at diagnosis (years), race (Black, Other, White, or missing), sex (male or female), population density (population/square mile), census tract median income ($), % population physically inactive, % of census tract that currently smokes cigarettes, and % of census tract that was up-to-date on colorectal cancer screening.

**Model 2 is adjusted for age at diagnosis (years), race (Black, Other, White, or missing), sex (male, female), population density (population/square mile), census tract median income ($), % population physically inactive, % of census tract that currently smokes cigarettes, % of census tract that was up-to-date on colorectal cancer screening, marital status (single, married, separated or divorced or widowed, and missing), family history of colorectal cancer (yes, no, or missing), health insurance (none, private, public, or missing), % of census tract who graduated high school, and % not in the labor force.
